# Internal Ribosome Entry Site (IRES)-Mediated Translation and Its Potential for Novel mRNA-Based Therapy Development

**DOI:** 10.3390/biomedicines10081865

**Published:** 2022-08-02

**Authors:** Rita Marques, Rafaela Lacerda, Luísa Romão

**Affiliations:** 1 Department of Human Genetics, National Institute of Health Dr Ricardo Jorge, 1649-016 Lisbon, Portugal; rita-r-marques@hotmail.com (R.M.); rafaela.santos@insa.min-saude.pt (R.L.); 2 BioISI—Biosystems & Integrative Sciences Institute, Faculty of Sciences, University of Lisbon, 1749-016 Lisbon, Portugal

**Keywords:** RNA-based therapies, internal ribosome entry sites, IRES *trans*-acting factors, antisense oligonucleotides, IRES-based multicistronic vectors

## Abstract

Many conditions can benefit from RNA-based therapies, namely, those targeting internal ribosome entry sites (IRESs) and their regulatory proteins, the IRES *trans*-acting factors (ITAFs). IRES-mediated translation is an alternative mechanism of translation initiation, known for maintaining protein synthesis when canonical translation is impaired. During a stress response, it contributes to cell reprogramming and adaptation to the new environment. The relationship between IRESs and ITAFs with tumorigenesis and resistance to therapy has been studied in recent years, proposing new therapeutic targets and treatments. In addition, IRES-dependent translation initiation dysregulation is also related to neurological and cardiovascular diseases, muscular atrophies, or other syndromes. The participation of these structures in the development of such pathologies has been studied, yet to a far lesser extent than in cancer. Strategies involving the disruption of IRES–ITAF interactions or the modification of ITAF expression levels may be used with great impact in the development of new therapeutics. In this review, we aim to comprehend the current data on groups of human pathologies associated with IRES and/or ITAF dysregulation and their application in the designing of new therapeutic approaches using them as targets or tools. Thus, we wish to summarise the evidence in the field hoping to open new promising lines of investigation toward personalised treatments.

## 1. Introduction

The use of RNA-based therapies started in the 1990s with a study in mice that proved the injection of a messenger RNA species (mRNA) in skeletal muscle led to the synthesis of the counterpart protein [1]. However, mRNA is but one of many forms of RNA in the cell, and the discovery of interference RNA (RNAi) granted the chance of repressing gene activity using short-interfering RNA (siRNA) in *Caenorhabditis elegans* [2]. Since then, several therapies based on the use of different kinds of RNA have been developed and approved. The first example of an RNA drug approved by the US Food and Drug Administration (FDA) is Fomivirsen, an antisense oligonucleotide (ASO) that inhibits human cytomegalovirus (CMV) and tackles CMV retinitis in human immunodeficiency virus (HIV) patients who do not respond to other treatments [3]. Recently, several other drugs have been developed and approved as therapies, such as Nusinersen, an ASO that corrects splicing defects associated with spinal muscular atrophy [4], or Patisiran, an RNAi therapeutic to treat hereditary transthyretin-mediated amyloidosis [5].

**Figure 1:**
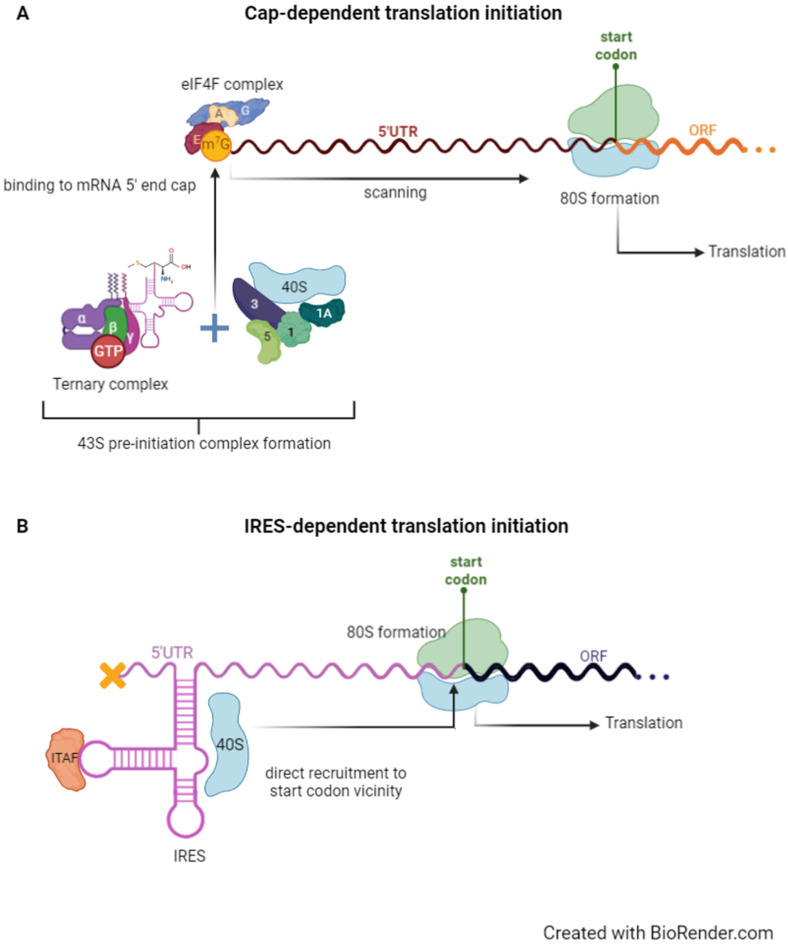
Cap-dependent versus internal ribosome entry site (IRES)-dependent translation initiation. (**A**): Canonical 5′ cap-dependent translation initiation. The canonical eukaryotic translation initiation depends on the recognition of the cap structure at the 5′ end of transcripts by the small ribosomal subunit (the 40S). The binding of several eukaryotic initiation factors (eIF1, eIF1A, eIF3, and eIF5) to the 40S subunit and the simultaneous formation of the ternary complex, composed of eukaryotic initiation factor (eIF) 2 bound to guanosine triphosphate (GTP) and initiator methionyl-tRNA, allows the assembly of the 43S pre-initiation complex (PIC). Simultaneously, a group of eIF4 factors is responsible for some of the interactions that eventually lead to mRNA activation. eIF4E binds to the 5′ cap and consequently to eIF4G, and eIF4A interacts with eIF4G:eIF4E, thus forming the trimeric eIF4F complex. Once these connections have been established, the 43S PIC binds to the cap structure at the mRNA 5′ end, becoming the 48S initiation complex, which in turn scans the 5′ untranslated region (UTR) until reaching the first initiation codon in a favourable context. After the codon recognition, 60S subunit joining and consequent 80S formation are induced, hence promoting further elongation and peptide synthesis. At this stage, eIF2 recycling is required to enable another round of translation initiation. (**B**): IRES-dependent translation initiation. This alternative mode of translation initiation does not need cap recognition nor the scanning of the 5′ UTR. Instead, there are some elements, the internal ribosome entry sites, which are intricate mRNA secondary structures usually located within the 5′ UTR of the transcript, which can directly recruit the 40S subunit to the vicinity of the initiation codon. This binding does not require complete assistance from eIFs, happening with the help of just a few eIFs or some IRES *trans*-acting factors (ITAFs), RNA-binding proteins that regulate IRES activity, either activating or repressing it. Once again, after the recognition of the initiation codon, both ribosomal subunits are assembled and ready for elongation, thus leading to peptide synthesis.

The use of RNA-based therapies to modulate protein expression is being deepened and the alternative modes of translation initiation are attractive targets and tools for pharmacological transformation. Canonical eukaryotic translation initiation depends on the recognition of the cap structure present in the 5′ end of mRNAs by the pre-initiation complex, and posterior 5′ untranslated region (UTR) scanning until reaching the first initiation codon in a favourable context (Figure 1A) [6,7]. Once there, the 80S ribosome is assembled, and peptide chain synthesis starts. However, under stress conditions, canonical translation initiation is impaired and global protein synthesis decreases, due to the triggering of an integrated stress response (ISR), whose main intrinsic factor is endoplasmic reticulum stress caused by the accumulation of unfolded proteins as a consequence of nutrient deprivation, hypoxia, oxidative stress, ultraviolet radiation, viral infection, inflammation, and others [7,8]. Eventually, the ISR leads to the expression of genes that fix cell damage or, if homeostasis cannot be resumed, to a cascade of events triggering apoptosis [8]. The expression of such genes can be maintained by alternative mechanisms of translation initiation, some of them independent of cap structure recognition and 5′ UTR scanning [6]. There are several described mechanisms of cap-independent translation initiation [6,9,10,11], and one of the most studied is the one mediated by internal ribosome entry site (IRES) elements. IRES-mediated translation (Figure 1B) consists of the recruitment of the 40S ribosomal subunit directly to the vicinity of the initiation codon, through an IRES element within the transcript 5′ UTR, bypassing the need for cap recognition or 5′ UTR scanning [12]. IRES activity can be assisted by IRES *trans*-acting factors (ITAFs), which are responsible for stabilising a specific IRES conformation, thus allowing the binding of the small ribosomal subunit directly to the mRNA [6,10,13]. ITAFs are key regulators that control IRES-dependent translation initiation, either by promoting or inhibiting IRES activity, playing a vital role in the cell response to stress conditions and many other physiological processes, including cell differentiation and proliferation, migration and invasion regulation, cell cycle progression, or apoptosis [7,14]. There are around 50 proteins that have been associated with the regulation of cellular IRES activity, either through activation or inhibition [7]. Dysregulation of ITAFs, and, hence, of IRES-mediated translation initiation, has been shown to promote the expression of many oncogenic mRNAs [15]. Besides cancer, IRES-mediated translation initiation and ITAFs have been strongly linked to other pathologies, such as cardiovascular diseases, neurodegenerative diseases, muscular atrophies, and other genetic diseases [7]. Although the information about the participation of IRESs and ITAFs in cancer and corresponding therapies [15,16] is a growing field, regarding other pathologies, the information about IRES- and ITAF-based therapies is scarce and a valuable field in which to invest more. 

Here, we intend to review several groups of pathologies that have been associated with IRES and/or ITAF dysregulation and the existing knowledge about how they can be used, either as targets or tools, to design new therapies. By doing so, we aim to summarise the current information in the field to understand what is missing, so that new lines of investigation on the treatment of such diseases can be developed.

## 2. Cap- Versus IRES-Dependent Translation Initiation

Cap-dependent translation initiation requires the recognition of the cap structure at the 5′ end of transcripts by the small ribosomal subunit (the 40S) and several eukaryotic initiation factors (eIFs), followed by 5′ UTR scanning until an initiation codon in a favourable context is recognised, whereas IRES-dependent translation initiation depends on direct binding of the 40S to the vicinity of the initiation codon with little or no dependence on eIFs nor 5′ UTR scanning [6,7]. 

### 2.1. Canonical 5′ Cap-Dependent Translation Initiation 

Canonical translation initiation (Figure 1A) is a highly regulated process that depends on several eukaryotic initiation factors and the dynamic and complex interactions among them [6]. In eukaryotes, the canonical translation initiation depends on the addition of a 7-methylguanosine cap (m^7^G) at the 5′ end of mRNA, due to the cleavage of the γ-phosphate of the mRNA 5′ end, and consequent GMP transfer from GTP through guanylyltransferase and its N^7^-methylation by (guanine-N7-)-methyltransferase [17]. The cap structure is crucial to protecting mRNA from 5′ to 3′ exonuclease degradation, and also accounts for its recognition and translation, serving as a molecular tag [9]. Cap-dependent translation initiation requires the 40S ribosomal subunit to bind the 5′ end cap structure, alongside several eukaryotic initiation factors (eIFs). Initially, the ternary complex is formed, in which eIF2 binds to GTP and initiator methionyl-tRNA (Met-tRNA_i_^Met^), an interaction regulated by eIF2 subunits. Then, the 43S pre-initiation complex (PIC) is formed as a result of the binding of the ternary complex to the 40S ribosomal subunit with the help of eIF1, eIF1A, eIF3, and eIF5. At the same time, eIF4F is responsible for activating the mRNA and the mRNA interaction with the ribosome. eIF4F is composed of eIF4E, eIF4A, and eIF4G. eIF4E binds directly to the 5′ cap and, subsequently, to eIF4G; then, eIF4A (an ATP-dependent RNA helicase) interacts with eIF4G:eIF4E, forming the trimeric eIF4F complex [6,18]. The affinity of eIF3 to eIF4G allows the 43S PIC to be recruited and attached to the cap-proximal region of the previously activated mRNA [19] to form the 48S initiation complex, which scans the 5′ UTR until reaching the first initiation codon (usually AUG) in a favourable context. The scanning process is ATP-dependent and requires RNA helicase activity and eIF4H RNA-unwinding activity, to allow the translocation of the 48S in a 5′ to 3′ direction [6,9]. After start codon recognition, the scanning factors dissociate and the Met-tRNA_i_^Met^ binds to eIF5B:GTP [20]. Once in the ribosomal P-site, the AUG codon pairs with the corresponding anticodon triplet of the transfer RNA (tRNA). Then, eIF2-GTP is hydrolysed by eIF5B to promote 60S subunit joining and further 80S ribosome formation [6]. Since the released eIF2 is connected to GDP, it must be recycled and bound to GTP again, in an interaction highly regulated by eIF2B, to form a new ternary complex and start a new round of translation initiation, being one of the limiting steps of this phase [6,9].

Stress conditions can inhibit cap-dependent translation initiation in two main ways, which are either eIF2α phosphorylation or hypophosphorylation of 4E-binding proteins (4E-BPs) induced by the mechanistic target of rapamycin (mTOR) kinase inactivation. On the one hand, due to the high affinity between phosphorylated eIF2α and eIF2B, there is the formation of a tight eIF2α-P/eIF2B complex that reduces eIF2B availability and its further binding to eIF2. Thus, GDP–GTP exchange on eIF2 does not occur and, consequently, the eIF2-GTP-tRNA_i_^Met^ complex is not formed, which inhibits the binding of the 40S subunit and further canonical translation initiation [21]. On the other hand, the hypophosphorylated 4E-BPs have a higher affinity to eIF4E than the latter’s to eIF4G, so eIF4E does not bind to eIF4G and eIF4F is not assembled, which inhibits cap recognition and further cap-dependent translation initiation [22]. 

### 2.2. IRES-Dependent Translation Initiation and Its Regulation by IRES Trans-Acting Factors 

IRES structures were discovered in poliovirus mRNAs [23]. Most viral mRNAs are uncapped and, therefore, they rely on IRES elements within their 5′ UTRs to recruit the ribosome and initiate translation [24]. Viral IRESs are well-characterised and grouped according to their secondary structure and ITAF dependence [25]. On the other hand, cellular transcripts are capped and so their IRES-dependent translation is mostly triggered under stress conditions that impair canonical translation [24]. About 10 to 15% of cellular mRNAs can be translated by an IRES-dependent mechanism (Figure 1B) [6,26], although only nearly 100 transcripts have been reported to contain IRES elements [27]. The IRES-containing mRNAs are usually canonically translated and only switch to IRES-dependent translation under stress conditions. Thus, to cope with the translational switch during stress, cellular IRESs contain a less complex structure than their viral counterparts, and can even be modular, i.e., the IRES activity is scattered all through the 5′ UTR [28]. IRES activity is often regulated by aiding factors, the ITAFs, that can remodel IRES structure and activity [29] by contributing to stabilising IRES structures or to inducing conformational changes that allow or inhibit ribosome recruitment and its further correct positioning [24]. Some ITAFs are common to different IRES elements, such as the polypyrimidine track-binding (PTB) protein 1, which interacts with several IRES structures, leading to IRES activation or inhibition, under different stress conditions [7]. On the other hand, the same IRES can be regulated by different ITAFs, as is the case of the *p53* mRNA IRESs [30]. In normal conditions, the two IRESs regulating the expression of two p53 isoforms, the full-length and the shorter ∆40p53 isoform, are inhibited by two ITAFs—programmed cell death protein 4 (PDCD4) and nucleolin. Under stress conditions, different ITAFs bind to the IRESs and enhance their activity—ribosomal protein (RP) L26 and heterogeneous nuclear ribonucleoprotein (hnRNP) Q, for the full-length isoform; and PTB, death-associated protein 5 (DAP5), PTB-associated splicing factor (PSF), and Annexin A2, in the case of ∆40p53 isoform [7,30]. ITAF assistance is not the only mechanism to regulate IRES-mediated translation. Interaction of IRES elements with other *cis*-acting elements within the 5′ UTR, such as upstream open reading frames (uORFs) or RNA G-quadruplexes (RG4s) can modulate IRES activity [24]. The zipper model of translation control proposes that the translation of a uORF remodels the mRNA structure and promotes a shift to a translationally active IRES structure, as is the case of the transcript encoding the *amino acid transporter, cationic 1* (*CAT1*) [31,32]. There are some examples in which uORFs repress IRES activity, as is the case of *vascular endothelial growth factor* (*VEGF*) *A* and *fibroblast growth factor* (*FGF*) *9*. As for *VEGF-A*, there is a uORF within the IRES that is internally translated and blocks the main ORF IRES-mediated translation [33]. Regarding *FGF9* mRNA, there is a uORF upstream of the IRES structure that represses FGF9 protein synthesis in normal conditions, but not under hypoxia, leading to a switch to IRES-dependent translation and concomitant increase in FGF9 protein levels [34]. Thus, cellular IRESs can be grouped according to their characteristics and interactions: (i) assisting ITAFs able to alter IRES structures; (ii) uORFs that sequester ribosomes and modify IRES structures; and (iii) other structures existing within IRES structures (reviewed in [24]). 

#### 2.2.1. IRES-Dependent Translation of Circular RNAs (circRNA)

circRNAs are generated from mRNAs via spliceosome-mediated back-splicing, in which the 3′ splicing site is covalently linked to the 5′ splicing site [35,36,37,38]. circRNAs can consist of exons or introns and, given their closed-loop structures, are less prone to exonuclease degradation, compared to linear RNAs [37]. circRNAs are widely distributed in the organism, strongly tissue-specific, and have multiple functions, including the regulation of alternative splicing and transcription, binding to proteins, sequestration of proteins, as in the case of miRNAs, and interaction with RBPs, meaning they are important regulators of gene expression [39]. circRNAs are extremely stable and accumulate over time, that is, long-lived circRNAs may act as a repository for translation, which may be useful in case of physiological changes or stress responses [39]. Besides their non-coding functions, circRNAs can also be translated into proteins through cap-independent mechanisms, given the lack of the 5′ cap structure [36,38]. Indeed, Chen and colleagues successfully identified 71 out of 119 reported IRES sequences as being present in circRNAs [35]. These authors also showed that IRES-mediated translation of circRNAs can be facilitated by AU-rich sequences, 18S ribosomal RNA (rRNA) complementarity and a distinct secondary structure (SuRE) present on the IRES [35]. The same study also revealed that many IRESs on circRNAs are located near the back-splicing junction (BSJ) and that the recruitment of ITAFs could depend on this structure or the circRNA-specific nuclear export pathway [35]. Other mechanisms, such as RNA methylation patterns on the circRNA near the BSJ, also appear to regulate circRNA-specific IRES activity [40]. Nevertheless, circRNAs can also present an N^6^-methyladenosine (m^6^A) modification that mediates per se cap-independent translation [37,41]. Thus, circRNA translation can be initiated by IRESs or m^6^A-modifications [35,36]. In circRNAs IRES-mediated translation, elF4G2 directly binds to the IRES and recruits the 43S complex to initiate translation. When circRNAs do not contain an IRES, usually there is an m^6^A modification, which is recognised by RNA binding proteins, like the YT521-B homology (YTH) domain family protein (YTHDF) 1 or 3, which can recognise m^6^A and recruit eIF4G2 to initiate translation. Since circRNA-encoded proteins are usually truncated versions of the linear mRNA-encoded ones, such proteins may present similar functions or compete with the proteins produced from the linear mRNA [39]. It appears that peptides/proteins encoded by circRNAs may have a fundamental biological role and present substantial clinical significance [37,38] and important biological functions, as they associate with the regulation of cell proliferation, differentiation, migration, and myogenesis [35]. Furthermore, evidence has shown that dysregulation of circRNAs expression is closely correlated with various pathologies, such as Alzheimer’s disease, osteoarthritis, diabetes, cardiac diseases, and cancer [27]. circRNAs have been highly associated with cancer initiation, development, and drug resistance, being mainly responsible for the expression of oncogenes [39]. Also, circRNAs can be involved in a tumour microenvironment through intercellular communication due to their abundance in exosomes and human fluids, being clinically significant and promising biomarkers for cancer [39]. Actually, several translated circRNAs have been identified as pivotal in human cancer development and progression, specifically in glioblastoma, breast cancer, and colon cancer [37,38]. Other circRNAs encode peptides with significant antitumour functions by interfering in cancer metabolic reprogramming or metastasis [38]. Examples include circFGFR1p, a protein encoded by *circFGFR1* (circular FGFR1 mRNA), which functions as a negative regulator of FGFR1 oncoprotein to suppress cell growth during stress conditions, but is down-regulated in cancer cells and promotes an increase of the proliferative signalling [35]; *circSHPRH,* a circRNA encoding the SHPRH-146aa peptide, which suppresses tumorigenesis in glioma, while it can function as a miRNA sponge to inhibit hepatocellular carcinoma progression; and *circZNF609*, which also acts as a miRNA sponge to promote breast cancer progression and encodes a protein important in myogenesis [38]. m^6^A modifications have also been shown to play critical roles during normal brain development and function, and hematopoiesis [42]. Accordingly, they have also been implicated in other human pathologies, including psychiatric disorders, metabolic syndromes, and cardiovascular diseases, such as cardiac hypertrophy, heart failure, ischemic heart disease, and pulmonary hypertension [42]. Regarding the interaction between circRNAs and m^6^A modifications, circRNAs have shown to regulate the proliferation, metastasis, stemness, and resistance to therapy of non-small-cell lung cancer (NSCLC) and dysregulated m^6^A profiles have been implicated in the carcinogenesis and progression of NSCLC [41]. Methyltransferase like 3 (METTL3), a component of the methyltransferase complex that catalyses N^6^ methylation, is elevated in NSCLC and facilitates NSCLC metastasis by promoting the translation of m^6^A-modified YAP. However, the specific function of m^6^A modification and m^6^A-modified circRNAs in regulating the antitumour immunity of NSCLC remains elusive [41]. In sum, targeting m^6^A RNA modification factors could provide potential therapeutic strategies for various human cancers [42]. These recent findings give a new perspective on the research on circRNAs and suggest new circRNA-encoded proteins will be discovered soon, not just with an important role in carcinogenesis, but also with possible links to other pathologies [37,38]. However, there is still much to understand about the translation process of circRNAs and the mechanisms of their regulation. With the development of RNA deep sequencing techniques, more circRNAs can be validated as important therapeutic tools and targets, given that the circRNA-translated peptides/proteins might be useful as specific biomarkers for diagnosis, intervention, and prognosis [36,37]. Since their translation is mainly IRES-mediated, knowing their ITAFs and mechanisms of action will open new avenues to develop new therapeutics to treat conditions associated with abnormal circRNA activity.

#### 2.2.2. IRES-Mediated Translation of Different Protein Isoforms from Monocistronic Genes

Most cellular mRNAs are monocistronic, contrary to their viral counterparts, which are often bi- or polycistronic [43]. Some monocistronic mRNAs can encode more than one protein from the same transcript, thanks to alternative initiation codons scattered through out the coding region downstream of the first one, originating different N-terminally truncated proteins with the same C-terminal region. An example is the *p53* gene, which encodes several protein isoforms as a result of alternative translation initiation [44,45,46]. Besides the full-length isoform, whose translation can be either cap-dependent or IRES-dependent, some of the N-truncated shorter p53 isoforms can also be translated through IRES elements located within the coding region of the full-length *p53*, upstream of the corresponding initiation codon, as is the case of ∆40p53 [47]. Other instances include the angiogenic growth factors FGF2 and VEGF-A [7,48,49]. Also, *FGF2* mRNA contains one AUG and four CUG codons used to express five different isoforms with specific roles and locations, of which translation from the further upstream CUG is cap-dependent, whereas translation from all the remaining start codons is IRES-dependent [48,49]. As for *VEGF-A*, there are two IRESs within its mRNA that drive translation from a CUG and an AUG and lead to the synthesis of different isoforms with different cellular locations [49,50]. Altogether, these examples show the importance of IRES-mediated translation to enhance the post-transcriptional variability needed to rapidly respond to sudden environmental changes, so cells can adapt to and recover from stress conditions that cause the development of many diseases.

#### 2.2.3. IRES-Mediated Translation of Polycistronic Genes

Several polycistronic transcripts have already been identified in mammals, whose translation of the downstream cistron(s) is IRES-dependent [43]. Based on mRNA structure and function of gene products, mammalian polycistronic genes may be grouped in five distinct categories, as described below:

(i) a single transcript that coordinately expresses at least two protein subunits that are part of a multi-subunit complex. This is the case of tenocyclidine [1-(1-(2-thienyl)cyclohexyl)piperidine binding protein (TCP-BP), which is present in rat brain synaptic membranes and binds glutamate agonists. It is composed of two subunits, PRO-1 and PRO-2; the former is cap-dependently translated, whereas the latter is IRES-dependently translated through an element occurring in the intercistronic region [43,51];
(ii) a single transcript that encodes different protein products with similar structure and function that are differentially expressed, i.e., transcripts that include two cistrons, one encoding a primary protein expressed through a cap-dependent translation mechanism and another encoding a secondary protein translated through a cap-independent mechanism. An example of such a transcript is the free fatty acid receptor 1 (*FFAR1*), which encodes the G-protein receptor (GPR) 40 and the GPR41 [52]. GPR40 is a receptor for long chain fatty acids, whereas GPR41 is activated by short chain fatty acids [43,53]. This cistronic organisation accounts for coordinated regulation of both receptors. Another example that fits in this group is the *meloe* mRNA. This is a polycistronic transcript responsible for expressing the melanoma antigens MELOE-1 and MELOE-2, which contain functional IRESs to mediate the expression of such proteins [54]. IRES-mediated translation accounts for the selective expression of these two proteins in melanoma cells, rather than in normal melanocytes [54]. Charpentier et al. identified MELOE-3, a protein with poor immunogenicity encoded by an additional ORF in the 5′ UTR of *meloe* and translated by the cap-dependent mechanism, reinforcing the importance of targeting MELOE-1 and MELOE-2 IRES-dependent translation for melanoma immunotherapy [43,55];
(iii) a single transcript that encodes functionally distinct proteins whose expression is programmatically related, meaning two proteins that function differentially but play a role in the same pathway [43], like the *PITSLRE/CDK11* duplicate genes *CdcL1* and *CdcL2*. Each one encodes two cyclin-dependent protein kinase isoforms, p110 and p58, of which the p58 is IRES-translated [56]. This IRES-dependent translation is cell cycle-dependent and allows translation of p58 during the G2/M transition [43,56]. Also, the voltage-gated Ca^2+^ channel (*CACNA1A*) mRNA is bicistronic and encodes both the normal-length α1A subunit (wild-type transcription factor α1 antichymotrypsin, α1ACT) and the expanded polyQ tract subunit (extended α1ACT). The latter is an IRES-translated protein from at least one spliced form of the same *CACNA1A* mRNA [57]. The myotrophin (*MTPN*) gene is also transcribed into an mRNA with two adjacent tandem ORFs. These ORFs express two proteins—myotrophin, translated through the cap-dependent mechanism, and autosomal dominant adult-onset distal myopathy-6 (MPD6), translated through an IRES element [58]. Similar to what happens to *CACNA1A*, the proteins encoded by *MTPN* have distinct roles, but are programmatically related [43]—myotrophin works in the dimerization of NFκB in cardiac tissue and MPD6 is associated with the immune response in some types of cancer [58];
(iv) a single transcript that encodes proteins produced by stimulus-coupled protease cleavage or by IRES-dependent translation initiation [43]. This is the case of transcripts with two overlapping ORFs that code products required for signal transduction, in which the first cistron codes for a receptor initiating signal transduction upon ligand binding, whereas the downstream cistron produces a constitutively active signal [43]. *Notch2*, for instance, is a gene encoding a receptor involved in the ligand-receptor notch-signalling pathway [59]. The interaction of Notch2 with the extracellular notch ligand triggers the protease cleavage of the C-terminal polypeptide, the notch intracellular domain (NICD) [60]. Notch2-ICD is translated via an IRES occurring in the *Notch2* coding region [60]. Another example is the *Her2* gene, a tyrosine kinase receptor involved in cancers and neurodegenerative diseases. It is a polycistronic gene encoding the full-length HER2 protein and several C-terminal fragments (CTF) [61]. These CTFs are translated through IRESs within the *HER2* coding region [43,61];
(v) a single transcript with different ORFs separated by IRES-containing intercistronic regions. This is the case of the tricistronic *c-myc* mRNA that, when transcribed from the alternative upstream promoter P0, contains three different ORFs separated by two intercistronic regions each containing an IRES [62,63]. The two identified IRESs mediate the translation of both the second and third ORFs that encode the MYCHEX1 and c-myc1/c-myc2 proteins, respectively [63].

## 3. IRES-Dependent Translation Dysregulation-Related Diseases

IRES-dependent translation in humans, either of mono- or polycistronic transcripts, is associated with many diseases. Cancer is, by far, the most well-characterised set of conditions affected by IRES or ITAF dysregulation. However, it is widely appreciated that dysregulation of IRES-mediated translation is also associated with other pathologies. Here we provide a few examples of diseases, other than cancer, whose aetiology relies on the dysregulation of translation initiation and the related IRESs and ITAFs. 

### 3.1. Neurodegenerative Diseases

#### 3.1.1. Spinocerebellar Ataxia Type 6 (SCA6)

Spinocerebellar ataxia type 6 (SCA6) is an autosomal dominant inherited neurodegenerative disease, presenting an incidence of about 5/100,000 persons. SCA6 is a late-onset progressive disease, in which the patients present progressive cerebellar ataxia and atrophy, and simultaneous selective Purkinje cell degeneration typically developed from the age of 40 [64]. SCA6 is caused by the polyQ expansion in α1ACT, which is translated through an IRES upstream of the second cistron of the *CACNA1A* gene. It was demonstrated that the elimination of the *CACNA1A* IRES sequence led to the abolition of the expression of the SCA6-associated α1ACT (α1ACT_SCA6_) protein. Mutated mice with the extended α1ACT presented a considerable reduction of the molecular layer thickness and a 50% loss of Purkinje cell dendritic tree density, which correspond to pathological features of SCA6 [64]. Since total silencing of *CACNA1A* gene expression would be lethal, selective elimination of α1ACT expression could be a safer therapeutic attempt for SCA6 [65]. For example, a *CACNA1A* IRES-targeted therapeutic method, using the expression of specific miRNAs, could offer a better approach for treating SCA6 [65,66]. The miRNA specifically interacts with *CACNA1A* IRES through the predicted binding site and inhibits α1ACT IRES-driven translation, without impairing *α1A* expression and *CACNA1A* mRNA expression [64]. Results have shown that the treatment promotes the protection of the Purkinje cells from degenerative changes, by inhibiting the degeneration caused by *CACNA1A* IRES-driven α1ACT_SCA6_ [64]. Additionally, the mice also exhibited an improvement in gait instability in all four limbs and avoid weaving movement, performing significantly better. In conclusion, these studies proved that the directed RNA-based therapy to selectively prevent *α1ACT* IRES-mediated expression could be used to treat SCA6 [64]. In the future, the use of ASOs and RNAi approaches would also be promising strategies to target and modulate *α1ACT* IRES-mediated expression [67].

#### 3.1.2. Fragile X Syndrome

Fragile X syndrome (FXS) causes intellectual disability and autism. It is the most common hereditary neurological condition and is a consequence of the lack of fragile X mental retardation protein (FMRP) [68]. FMRP is an RNA-binding protein present in the brain, responsible for controlling the translation of several neuronal mRNAs and synaptic functions and structures [69]. While healthy individuals present about 30 repeats of CGG in the 5′ UTR of the fragile X mental retardation 1 (*fmr1*) gene, patients with FXS have over 200 repeats of CGG, which promotes the sequence hypermethylation. This causes the transcriptional inhibition of the *fmr1* gene and consequent absence of FMRP, promoting an impairment of synaptic responses [70]. It was proven that *fmr1* translation uses both cap-dependent and IRES-mediated mechanisms, as it contains an IRES in its 5′ UTR. Also, it was shown that *fmr1* IRES-mediated translation occurs with the involvement of the hnRNPQ as ITAF [70]. However, little is known about the effect of this mechanism on FXS development. In neuron development, the axonal growth cone of a neuron travels large distances to connect to the dendritic spine of the next neuron, depending on axonal guidance cues like semaphorins to direct the appropriate connection. In semaphorin 3A (Sema3A) treatment, a neuronal repellent that induces growth cone collapse, hnRNPQ synthesis in primary hippocampal neurons increases, which, in turn, when up-regulated, leads to Sema3A-induced growth cone collapse and consequent FMRP synthesis. Depletion of *fmr1* expression by siRNA, under treatment with Sema3A, prevented axonal growth cone collapse, which is also attenuated by reducing hnRNPQ expression. It was demonstrated that hnRNPQ over-expression restores IRES-mediated *fmr1* translation activity in hnRNPQ knockout cells, thus increasing FMRP expression. Thus, hnRNPQ acts as an ITAF that activates IRES-mediated *fmr1* translation, contributing to restoring FMRP levels, and, simultaneously, participates in Sema3A-induced axonal growth cone collapse [71]. Considering this dual effect, the role of *fmr1* IRES-mediated translation and its ITAF regulation on both conditions and associated pathologies is crucial in the development of novel specific therapeutic approaches [70]. Also, there have already been described several mRNA targets of FMRP, which could be used to develop new therapies, taking into account that the expression of FMRP is modulated by m^6^A modifications that can disrupt its binding to the respective targets [72].

#### 3.1.3. Alzheimer’s Disease

Alzheimer’s disease (AD), a neurodegenerative disorder, is the most frequent type of dementia in the elderly. Autosomal dominant inherited forms of AD correspond to no more than 5% of the cases, the remaining of sporadic origin. The disease is characterised by the accumulation of extracellular amyloid-β (Aβ) plaques, which consist mostly of Aβ peptide precursor protein (APP), and an increase in the aggregation of tau in neurofibrils within neurons [73]. APP is a type I membrane protein encoded by the *APP* gene, which presents more than 25 pathogenic mutations, all causing an autosomal dominant form of AD, hence being strongly linked to the pathogenesis of AD. Data have shown that APP over-expression leads to an increase in full-length and truncated p53 (p53 and p44, respectively) expression levels in the brain tissue [74]. This leads to cognitive decline and synaptic and memory defects [74]. By analysing the levels of p44 in mice brains, it was observed a consistent and statistically significant increase in the levels of p44 when APP was over-expressed. However, no change in p44 levels was observed when APP was lacking, suggesting that APP induction of p44 expression is not required for p44 baseline levels [74]. Amyloid precursor protein intracellular domain (AICD), the cytosolic tail of APP, binds to the *p53* IRES and regulates the translation of p44. When APP is over-expressed, mice rapidly develop AD-like neuropathology, indicating a possible link between ageing and AD [74]. Also, transgenic mice over-expressing AICD developed some of the features that characterise AD, such as abnormal activation or phosphorylation of tau kinases, synaptic deficits, and higher neuronal susceptibility to exogenous stress [74]. Reports have shown that patients with late-onset AD express increased levels of p44 [74,75]. Several proteins have been proven to be ITAFs of *p53* and to regulate the translation of p53 isoforms, as is the case of nucleolin and PTBP1. While nucleolin seems to produce a negative effect on p53 translation and decrease it in an age-dependent manner in the brain, AICD appears to be a positive factor [74]. So, there is a connection between AICD and p44 that may be involved in AD and other age-related tauopathies. Indeed, the role of p44 in longevity and cognitive-related events is complex requiring further studies. Further research has shown that m^6^A levels are also decreased in AD brains, as a consequence of significantly reduced expression of METTL3 [76]. A natural product and small-molecule inhibitor of fat mass and obesity-associated protein (FTO) demethylase, rhein, can partially rescue this scenario, therefore having a promising therapeutic use [76]. There have been described several circRNAs and long non-coding RNAs dysregulated in AD patients [77], but their relationship with IRES-mediated translation initiation is yet to be clearly understood. However, they have the potential to become promising therapeutic targets to address such diseases [78]. 

#### 3.1.4. Parkinson’s Disease

Under hypoxia, the major transcription factor hypoxia-inducible factor (HIF)-1α binds to hypoxia-responsive elements (HREs) in the promoter to up-regulate HRE-containing genes [79]. This event regulates several cellular processes, like glucose metabolism, biosynthetic pathways, cellular metabolism reprogramming and cell viability [80,81]. There are numerous lines of evidence linking HIF-1α to Parkinson’s disease (PD) [82]. PTEN-induced putative kinase-1 (PINK1) is a serine/threonine kinase that has several distinct functions on the mitochondria and cytosol, promotes cell survival, and activates the HIF-1α pathway. PINK1 mutations that contribute to protein instability or decreased kinase activity are linked to autosomal recessive Parkinson’s disease. In the absence of wild-type PINK1, HIF-1α protein induction under hypoxia is reduced and cells present bioenergetic and mitochondrial unbalances, which are present in both sporadic and genetic forms of PD [82]. It seems that PINK1 exhibits protective effects against various oxidative stresses and facilitates stress response, due to the activation of 4E-BP1 and consequent up-regulation of IRES-dependent translation. On the contrary, in PINK1 deficiency, over-expressed 4E-BP1 fails to up-regulate IRES-dependent translational activity significantly. It was shown in some experiments that 4E-BP1 over-expression rescued the PINK1 deficient phenotype [82]. Additionally, HIF-1α and its targets are required to preserve dopaminergic neuron integrity, which might explain how HIF-1α loss can promote neurodegeneration in PD. The connection between translation and PD was also strengthened by a study that linked mutations in eIF4G1 with a familial case of PD and by the findings that 4E-BP1 may be a leucine-rich repeat kinase 2 (LRRK2) target, which is another protein associated with PD [82]. In conclusion, PINK1 is an important regulator of translation during stress response and an activator of the HIF-1α pathway, promoting the maintenance of energy metabolism and cell survival. It is yet to discover the role of HIF-1α and protein translation in PD pathogenesis, which will lead to better development of neuroprotective strategies. On the other hand, since alterations in the gene encoding α-synuclein (aSyn) protein can cause or increase the risk of developing PD, Cole et al. tested ASOs targeting the corresponding mRNA and observed inhibition in the protein synthesis that reverted the phenotype in rodent pre-formed fibril models of PD [83]. This supports the further use of strategies to correct the expression of mutated PINK1 ITAF and, thus, restore its protective effect.

#### 3.1.5. Amyotrophic Lateral Sclerosis and Other Neurological Conditions

hnRNPA2/B1, hnRNPA1, and fused in sarcoma (FUS) are RBPs often associated with neurological diseases and, therefore, it is of the utmost importance to maintain RBP physiological levels in the nervous system. Patients with motor neuron disorders, such as amyotrophic lateral sclerosis (ALS), and spinal muscular atrophy (SMA), among others, present mutations in genes encoding these RBPs. ALS is the commonest motor neuron disorder in adults, causing a gradual loss of upper and lower motor neurons and, eventually, fatal paralysis. The causes of ALS are yet to be understood, whereas most cases are sporadic and only 10% are hereditary [84]. There is a link between hnRNPA1 mutations and multisystem proteinopathies (MSP), a group of pleiotropic neurodegenerative disorders that includes ALS [85]. Furthermore, this protein, alongside hnRNPA2/B1, seems to be depleted in the brains of AD patients, and misfolding and fibrilization of this protein have been associated with the disease [86,87]. Decreased levels of hnRNPA2/B1 and hnRNPA1 in the entorhinal cortex of patients have been implicated in the pathogenicity of sporadic AD [84]. These data correlate cholinergic neuron loss with reduced hnRNP levels and missplicing, which might explain some cognitive deficits observed in AD. Mutations in FUS have also been linked to neurological diseases, such as frontotemporal lobar degeneration (FTLD), essential tremor, and Huntington’s disease [88,89]. It is important to notice that nearly every protein discussed above is involved in RNA transport and somehow in RNA splicing, thus, motor and nerve cells are also prone to be affected by dysregulations in these processes [84]. There is little information about the importance of IRES-mediated translation initiation in the development of these neurological conditions; however, the proteins related to such conditions act as ITAFs for a wide range of IRESs, meaning that their function may be compromised or dysregulated, either enhanced or inhibited and, for that reason, creating an imbalance in protein homeostasis that eventually affects the onset and development of the aforementioned conditions. Understanding the pathophysiological role of such RBPs will provide new prospects for research and the eventual development of RNA-based therapies targeting these diseases. 

### 3.2. Muscular Atrophies

#### 3.2.1. Ischemic Cardiomyopathy (Lymphangiogenesis Regulation)

Hypoxia is a key component of the tumour microenvironment and induces critical changes in tumour cell metabolism, angiogenesis and lymphangiogenesis [90]. However, hypoxia also constitutes major stress in other pathologies, such as ischemic pathologies, in which artery occlusion leads to hypoxic conditions, and then angiogenesis is promoted as a cellular response to fight the lack of oxygen and nutrients in cells. It has been shown that (lymph)angiogenesis is also induced by hypoxia and mediated at both transcriptional and post-transcriptional levels [90,91]. VEGF-A and FGF2, major angiogenic factors, and the lymphangiogenic growth factor VEGF-C are all induced by hypoxia through a translational mechanism [90]. As we have already mentioned above, several IRESs have been identified in the mRNAs of (lymph)angiogenic growth factors from the *FGF* and *VEGF* families, suggesting that the activation of angiogenesis and lymphangiogenesis during stress might be highly controlled via IRES-mediated translation [91]. VEGF-A has pro-lymphangiogenic properties and its induction under hypoxia occurs in both physiological states and pathological conditions, such as ischemia or tumour development [92]. During hypoxia, *VEGF-A* IRES activity is positively regulated by MAPK3 kinase and hnRNPL, and inhibited by DEAD-box RNA helicase 6, all acting as its ITAFs [90]. On the other hand, VEGF-C induces endothelial cell proliferation, migration, and survival, and, during tumour growth and under hypoxia in vitro, *VEGF-C* IRES activity was demonstrated to be up-regulated [90,93]. Furthermore, VEGF-D over-expression correlates with an increase in lymphatic vessel growth (tumour lymphangiogenesis) and lymphatic metastasis [90]. It appears to exist two waves of IRES activation in response to hypoxia: a first response phase corresponding to early hypoxia, in which IRESs from (lymph)angiogenic growth factor mRNAs are activated, while a second response includes “non-angiogenic” *c-myc* IRES, which is activated in late hypoxia [91]. Vasohibin 1 (VASH1), angiogenesis- and stress-related protein, has already been described for its expression in endothelial cells and HL-1 cardiomyocytes. *Vash1* mRNA translation is highly induced in early hypoxia and leads to a strong expression of VASH1, whose knockdown down-regulates earliest-induced IRESs, like *FGF1*, proposing this protein as a new ITAF in cardiomyocytes. Thus, under hypoxia, VASH1 is an activating ITAF of *FGF1* and *VEGF-D* IRESs, but in normoxia, it acts as an inhibitor. This suggests that VASH1 interacts with different partners in the IRESome or that exist different VASH1 isoforms, implying that the main response to early hypoxia in cardiomyocytes is at the translation level [91]. All these results are crucial for a better understanding of the acute stress response in the ischemic heart. Since the role of hypoxia in gene expression regulation has been mostly analysed in conditions of tumoral hypoxia, in which angiogenesis promotes the formation of abnormal vessels with a lack of function, it is important to study the response to hypoxia in the context of ischemic diseases. Indeed, HL-1 cardiomyocytes respond to hypoxia very early, whereas various human tumour cell lines require a longer time of exposure to hypoxia for IRES-dependent translation to be stimulated [91].

#### 3.2.2. Myogenesis Regulation

Although this is not a pathology per se, myogenesis regulation influences cell proliferation and differentiation of several cells, such as cardiomyocytes, due to different IRESs and ITAFs related to myogenesis, with further influence in cardiac diseases or cardiomyopathies. While FGF1 and FGF2 inhibit myoblast differentiation, FGF1 also activates such a process, thanks to a transcription-translation coupling mechanism [94]. There are four promoters (A, B, C, and D) that drive transcription of *FGF1*: while promoter A is specifically active in the heart, skeletal muscle, and kidney, and promoter B in the brain, C and D are inducible and related to cell proliferation. As an outcome of eIF4E sequestering by 4E-BP-1 [94], cap-dependent translation initiation is impaired during the early stages of myoblast differentiation, which results in the specific activation of *FGF1* IRES A through the myoblast differentiation process [95]. hnRNPM and p54^nrb^/NONO act together to activate *FGF1* IRES-mediated translation in a promoter-dependent manner, by binding to promoter A and IRES A [94,95]. This specific activation of *FGF1* mRNA accumulation and stability, due to hnRNPM and p54^nrb^ binding, is correlated with the induction of differentiation but is very weak during cardiomyocyte proliferation. p54^nrb^/NONO and hnRNPM are also important in myogenesis, as they are needed for myotube differentiation from myoblasts, which suggests that both proteins may work as activator ITAFs of *FGF1* IRES, despite no evidence of the direct interaction of p54^nrb^ and hnRNPM with the RNA [95]. All in all, FGF1 expression is controlled by the promoter and the translational regulating factors during myoblast proliferation and differentiation [95]. On the other hand, several circRNAs are involved in several muscular processes, such as myoblast proliferation and differentiation, and muscular development [96,97]. It remains to be deciphered the role of circRNA IRES-mediated translation in these muscular processes. This can be a great basis on which to develop therapeutic strategies for muscular disorders.

#### 3.2.3. Duchenne Muscular Dystrophy

Duchenne muscular dystrophy (DMD) is the most prevalent inherited neuromuscular disorder, with a prevalence of 1 in 3500 male births [98]. DMD develops due to deletions/mutations in the dystrophin gene, which prevents the production of full-length dystrophin molecules in skeletal muscle fibres. There have already been developed ASOs to correct splicing and restore the dystrophin levels in DMD patients [99,100]. Utrophin, the autosomal homologue of dystrophin, presents a high structural and functional similarity with the latter [101]. Utrophin A is the isoform expressed in skeletal muscles primarily in post-synaptic regions of the sarcolemma and its increased expression was identified in regenerating skeletal muscles. [102]. However, *utrophin A* mRNA levels did not increase concomitantly, suggesting that the increase in protein levels might be caused by changes in protein stability or translation efficiency, including the possibility of an IRES-dependent translation mechanism driving its expression [102]. Using bicistronic reporter vectors, it was demonstrated that the *utrophin A* 5′ UTR demonstrated no IRES activity in intact muscles. Indeed, the 5′ UTR of *utrophin A* causes a translation inhibition in skeletal muscle fibres under control conditions, whereas in regenerating muscles there is an IRES activation that accounts for utrophin A protein expression [103,104]. There is evidence that, in vivo, cap-independent translation driven by the *utrophin A* IRES occurs exclusively in skeletal muscles [102]. eEF1A2 (one of the two eEF1A isoforms) interacts with the *utrophin A* 5′ UTR in the same regions that can drive cap-independent translation in C2C12 myoblasts [102]. Mice that do not express functional eEF1A2 show motor neuron and muscle degeneration, which eventually leads to premature death [102]. However, eEF1A2 might not be the only protein required for skeletal muscle-specific *utrophin A* IRES activity [102]. For instance, FGF2 improves regeneration when injected into the muscles of mice, whereas insulin growth factor (IGF) 1 receptor expression is up-regulated in muscle regeneration. Of note is that *FGF2* 5′ UTR contains an IRES and IGF-1 translation is also IRES-dependent [16]. In this regard, muscle regeneration may be considered a “cellular stress” that promotes IRES-mediated translation [104]. Given all the data, the up-regulation of endogenous levels of utrophin in muscle fibres of affected patients could functionally outweigh the absence of dystrophin and, thus, be used as a possible DMD treatment [105]. Over-expression of utrophin in muscle fibres of a DMD mouse model has been shown to alleviate the dystrophic pathology, proving how the regulation of utrophin expression could contribute to important therapeutic advances [104]. 

### 3.3. Other Specific Diseases

#### 3.3.1. Diamond-Blackfan Anaemia

Diamond-Blackfan anaemia (DBA) is normochromic macrocytic anaemia characterised by the reduced erythroid precursors in the bone marrow, which is mostly diagnosed in infants less than one year of age, yet, recently, some cases have been diagnosed in adult patients [106]. About 50% of DBA patients have skeletal deformities, such as thumb malformations and growth retardation. In 55% of patients, DBA is associated with mutations in genes encoding ribosomal proteins, causing their haploinsufficiency and loss of function, ultimately impairing general translation [107]. An imbalance in the synthesis of ribosomal proteins activates *p53*, to which erythroblasts are extremely sensitive, inhibits cell proliferation, and may affect the translation initiation of specific transcripts important for erythroid differentiation, suggesting that DBA-associated severe anaemia is caused by a p53-dependent mechanism [107]. Knockdown of 40S ribosomal protein S19 (Rps19) in haematopoietic progenitors decreases the colony-forming capacity of erythroid progenitors, while in mouse foetal liver-derived erythroblasts impairs their proliferation, but not their differentiation [107]. Also, the knockdown of both Rps19 and Rpl11 resulted in phenotypical changes in erythroblasts during proliferation and differentiation [107]. Furthermore, reduced expression of Rps19 or Rpl11 repressed the translation of two essential transcripts for erythropoiesis, *Bag1* and *Csde1*, which are both translated from an IRES and tightly up-regulated in erythroid cells [107]. Csde1 is an RNA-binding factor that controls IRES-mediated translation, despite no regulation of *Bag1* mRNA [107]. Protein levels of Bag1 and Csde1 in erythroblasts from DBA patients are also low, although RNA expression is not affected [107]. The reduction of Csde1 expression inhibits both proliferation and maturation of erythroblasts, while the complete loss of Bag1 expression strongly impairs erythropoiesis and its reduction makes erythroblasts less prone to enter the terminal differentiation program [107]. This indicates that a reduction in Bag1 and Csde1 expression results in severe DBA due to a cooperative effect between them. All in all, the overall DBA phenotype seems to be caused by a combination of *p53* activation and a defective mRNA translation [107]. Since p53 translation may be mediated through an IRES, it is plausible to assume that dysregulation of p53 IRES-mediated translation accounts for the development of the disease. In this regard, new therapies targeting *p53* IRES would impair its translation and therefore reduce the erythroblast sensitivity to p53 activation. Following the same line, therapies modulating the expression of *p53* IRES regulatory ITAFs would contribute to regulating erythroblast sensitivity. 

#### 3.3.2. Diabetes

The insulin receptor (INR) and insulin-like growth factor (IGF) receptor pathways are essential for the evaluation and response to nutrient availability, also playing an important role in cellular proliferation regulation and cell size determination. Prolonged exposure of cells to insulin induces insulin receptors (INR) down-regulation via internalization and enhanced protein degradation, which causes an imbalance and can lead to type 2 diabetes in humans and other associated diseases [108]. It was confirmed the existence of a functional IRES on the *INR* 5′ UTR, which is strongly stimulated in the presence of PTB1 and nPTB, and slightly less stimulated in the presence of hnRNPK and PTB2. The 5′ UTR of *IGF-1R* mRNA also contains an IRES, as does the *Drosophila* insulin/IGF-like receptor (*dINR*) mRNA, which binds to HuR, a stability factor that inhibits translation, and hnRNPC, which enhances IRES-mediated translation and competes with HuR for the binding site [108]. There are differences in the location and sequence of the *INR* and *IGF-1R* IRESs, strongly suggesting different mechanisms of regulation, and distinct dependence on cell type and density [108]. Insulin itself could also stimulate IRES activity. Both INR and IGF-1R are expressed in the nervous system and have been correlated to important roles in neuronal development and protection from and/or promotion of age-related neurodegenerative diseases [108]. In *Drosophila*, the signalling cascade of insulin receptors activates the oncogenic protein kinase Akt, stimulating the modification and posterior phosphorylation of mTOR protein, which, in turn, inactivates the translation initiation inhibitor eIF4E-binding protein (d4E-BP) [109]. During high nutrient and high insulin-like peptide presence, d4E-BP is phosphorylated and inactive, unable to interact with eIF4E. This favours effective translation of many cellular transcripts no matter what mechanism of initiation is used. In contrast, in nutrient deficiency conditions or the absence of insulin, d4E-BP become dephosphorylated and active, inhibiting cap-dependent translation, and endorsing a selective translation of IRES-containing transcripts, such as *dINR* [109]. *INR* IRES may simply function to maintain the expression level of INR, fighting the inhibition of cap-dependent initiation under such conditions, including, perhaps, in some differentiated cells. However, in the case of the IGF-1R, there is evidence that regulation of expression does occur at a translational level [108]. All this information could provide new insight into insulin-resistant type 2 diabetes development. 

All the mentioned pathologies and their related IRESs and ITAFs are summarised in Table 1. By looking at the information gathered in the table, there is a missing link between the knowledge of IRES and their ITAF regulation and the use of RNA-based therapies to specifically target IRES elements or their regulating ITAFs. Again, the use of IRES-based vectors to express proteins that allow the rescue of some of the corresponding wild-type phenotypes is missing for many diseases other than cancer. 

## 4. RNA-Based Therapies to Modulate Translation Initiation Dysregulation

As we have just observed, there is little information regarding the therapeutical RNA-based approaches to treat diseases caused by dysregulation of translation initiation, especially those involving IRESs and ITAFs. RNA-based therapies are an emerging world of solutions to tackle several diseases [112]. An increasing number of yet-to-be-treated diseases originated from IRES-mediated translation dysregulation opens the way to explore such alternative modes of protein synthesis as promising therapeutic targets. From the point of view of influencing the activity of the IRES, therapeutical approaches modifying ITAFs activity, expression level, or IRES-ITAF interaction, may constitute important targeted therapies for many diseases or conditions. 

### 4.1. IRESs as Targets

**Figure 2:**
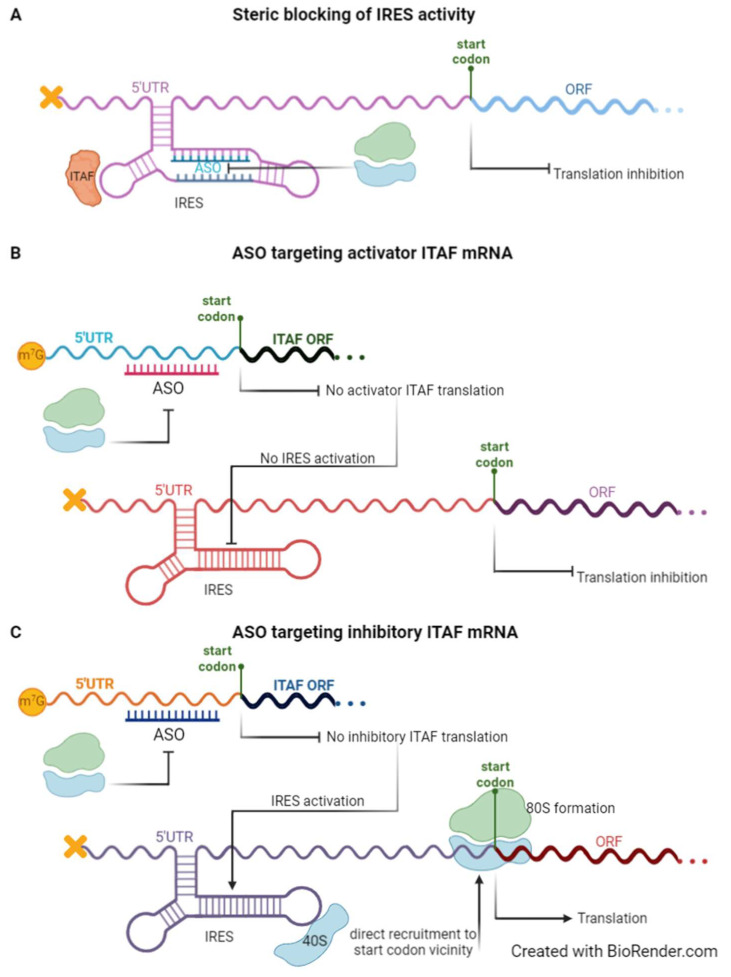
A model proposing the use of antisense oligonucleotides (ASOs) to modulate internal ribosome entry site (IRES) or IRES *trans*-acting factor (ITAF) activity and further protein expression. (**A**) An ASO targeting an IRES responsible for translating a pathogenic protein would lead to the disruption of IRES activity and hence hinder protein synthesis. (**B**) ASO targeting activator ITAF mRNA. If the ITAF enhances IRES activity, disrupting its expression would lead to impaired IRES activity and subsequent protein synthesis inhibition. (**C**) ASO targeting inhibitory ITAF mRNA. If the ITAF represses IRES activity, disrupting its expression would allow IRES activation and consequent regular protein synthesis.

The most widely used forms of drugs for medical use are small-molecule compounds and proteins, or antibodies, that act mainly on receptors, transporters, ion channels, kinases, and other enzymes [113]. The use of RNA molecules as new therapeutic approaches has emerged as a promising solution due to their specific and complementary physicochemical and pharmacological characteristics [113]. ASOs, siRNAs, miRNAs, guide RNA (gRNA), aptamers, or ribozymes, all work by different mechanisms, and their activity and pharmacokinetic properties can be independently optimised and consequently used to target IRES-mediated translation, thus becoming common therapeutic approaches. The use of such RNA molecules can result either in the destruction of the IRES structure or in the prevention of IRES interactions with ITAFs or the ribosome (Figure 2) [114]. Most RNA-based drugs currently used in the clinic or under development are oligonucleotide-based therapeutics. ASOs and siRNAs, although similar, as they both bind the target mRNA through Watson–Crick base pairing to block translation of the target protein, differ in their structure and mode of action; while siRNAs are double-stranded and cause the destruction of the target mRNA, ASOs are single-stranded and block translation, either by RNAse H-mediated cleavage or steric blocking of cellular factors [115,116,117,118]. Also, it is easier to deliver ASOs as they do not require a carrier, while siRNAs do, and simple chemical modifications allow greater resistance to nuclease degradation [116]. ASOs were the first type of nucleic acid drugs to be licensed and FDA has already approved some ASO-based drugs [119]. ASOs have already been used to prevent hepatitis C virus (HCV) IRES-mediated translation, by targeting and cleaving domain IIId of HCV IRES and, therefore, displacing the 40S ribosomal subunit [120,121]. Furthermore, the use of ASOs to target some 5′ UTR inhibitory elements, such as uORFs or RG4 structured regions, has already been reported as efficient in increasing the protein levels of human RNASHE1, LDLR, and ACP1, and mouse ACP1 and ARF1 proteins [122]. In this study, the authors showed an increased LDL uptake in cells treated with an ASO targeting *LDLR* mRNA, confirming the therapeutical potential of such an approach [122]. They delivered an ASO targeting a structured region within *ACP* 5′ UTR subcutaneously and observed increased protein levels in mice [122]. Thus, the same approach can be used to target structured cellular IRESs (Figure 2A). Even though many antisense oligonucleotide approaches have emerged as promising therapies to treat cancer [123], and the IRES mechanism has become a primary target for anticancer therapy [124], a combination of both, i.e., the specific targeting of IRESs or their regulatory ITAFs with ASOs, as a tool to treat cancer or, for that matter, the pathologies referred to in this review, is little explored. It is also possible to target ITAFs with specific ASOs that inhibit ITAF expression and, hence, modulate IRES activity depending on the effect of the ITAF on the regulated IRES—activator ITAF (Figure 2B) or inhibitory ITAF (Figure 2C). Several ASOs have already been designed to target cellular host factors needed to regulate IRES-mediated translation upon viral infection (reviewed in [125]). However, this approach could be more complex and less accurate since each ITAF can regulate more than one IRES simultaneously, creating a general effect on several pathways, instead of mediating a specific and desired interaction and/or IRES [114]. The fact that some ITAFs can have other roles in the cell also makes this approach less accurate. For example, the Staufen 1 protein, involved in the Staufen1-mediated mRNA decay mechanism [126] and differentially regulating growth, migration, and invasion in several prostate cancer cells [127] has recently been shown to act as an ITAF regulating HIV-1 IRES-mediated translation initiation [128]. Thus, altering the normal expression pattern of a given ITAF may result in detrimental effects in other cellular pathways, which means ASOs must be tissue- or even cell-specifically designed and their safety assessed [111]. On the other hand, some situations may benefit from a wider effect of the manipulation of ITAFs expression, but the conditions of each interaction and the specific effect on each IRES should be properly considered [16].

Larger RNA species, like mRNA, also present enormous therapeutic potential. Compared to DNA-driven or virus-based gene therapy, mRNA has distinct advantages: a higher penetrance rate in targeting cells, effectiveness in senescent cells, no integration into the genome, and consequent mutation development, since mRNA drugs are translated into proteins that can hence be targeted. Furthermore, in comparison to protein drugs, mRNA has a longer lifespan [113,129]. Since the size of the mRNA drug molecule is much bigger than other types of RNA therapeutics, there are delivery systems that allow an efficient mRNA direct administration to patients and additional protection from degradation by RNases and cross-cellular barriers in vivo [113,130]. 

**Figure 3:**
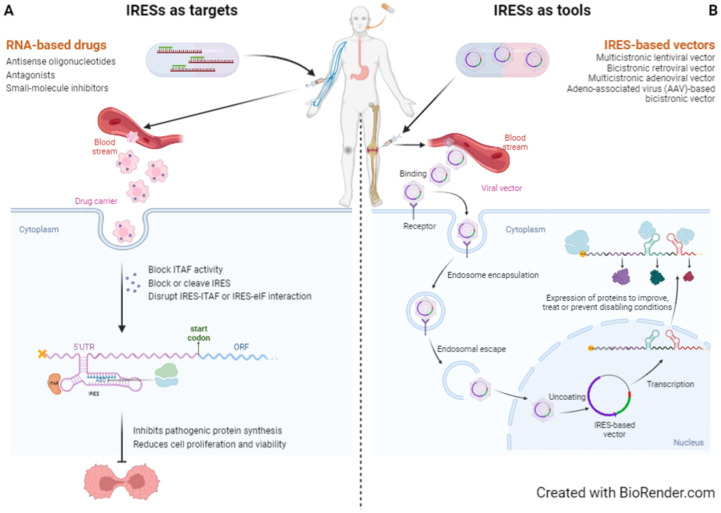
Mechanism of action of RNA-based therapies using internal ribosome entry sites (IRESs) as targets or tools. (**A**) IRESs as targets. RNA-based drugs, which can be composed of different RNA molecules, such as antisense oligonucleotides (ASOs), antagonists, or small-molecule inhibitors, are delivered into the bloodstream and carried to specific cells. Such RNA compounds bind to the mRNA and promote alterations in the IRES-mediated translation of pathogenic proteins, by blocking or cleaving the IRES structure, disrupting the interactions between the IRES element and both the ribosome and IRES *trans*-acting factors (ITAFs) and blocking the activity of ITAFs. These modifications lead to protein synthesis impairment, which may be crucial for the treatment or prevention of several pathologies, besides cancer. (**B**) IRESs as tools. Circular RNAs with IRESs, and several IRES-based viral vectors, have been used to produce non-pathogenic proteins with a therapeutic role. These vectors are injected into the bloodstream and then bound to specific cells. Once expressed, they can promote the simultaneous expression of more than one protein. The expression of these vectors allows for maintaining or enhancing the expression levels of proteins with important biomedical properties that present a positive effect on different conditions, constituting an important method of gene therapy and strategy of treatment for several diseases.

The most common drug-delivery systems used nowadays are polymer-based nanoparticles (LNPs), due to their ability to ensure adequate intracellular transportation, stability, and immune evasion while preserving similar efficacy and specificity. Besides, other materials, such as exosomes and quantum dots, offer new approaches to improve these drug-like properties of therapeutic nucleic acids [119]. When treating diseases that present no cure or difficulty in treatment and therapy resistance, such as several types of cancer or viral infections, resulting from translation dysregulation, including those mediated by IRES elements, mRNA-based therapeutical approaches have been proven as important tools [131]. Thereby, cellular IRESs represent an attractive novel therapeutic target [114]. One strategy of therapy is the use of antagonists, which can target specific RNA elements that control protein expression, such as IRESs (Figure 3A). Although the main aim of antagonists is the treatment of viral infections, these antagonists could also represent a new path for the prevention and treatment of other diseases, because they disrupt IRES interactions with the ribosome, the eIFs or ITAFs, by cleaving or blocking target IRESs [114]. Small-molecule inhibitors, one type of antagonist, have been shown to block the translation of IRES-containing transcripts, without blocking global cap-dependent translation [16]. These authors performed a high-throughput screening of 135,000 compounds to find out that three of them consistently and completely block *IGF1R* IRES-mediated translation initiation, and induce remarkable phenotypic alterations in human breast tumour cells [16]. Furthermore, there was a drastic loss of c-Myc in cells treated with the most promising identified IRES inhibitor compound—cpd_P [16]. Using *c-Myc* IRES, Didiot et al. identified a set of cardiac glycosides that inhibit IRES-dependent translation [132]. Their results showed that treating c-Myc-dependent cancer cells with such compounds leads to a reduction in c-Myc protein associated with a significant modulation of cell viability in ovarian cancer [114,132]. The study of these compounds and their mechanism of action would be useful to discover important signalling mechanisms and selectively perturb IRES-dependent translation, enabling the investigation of its contribution to physiological processes and pathological states and, ultimately, to discovering clinical applicability [15,16]. There are encouraging findings in the development of small-molecule inhibitors of the interaction between IRESs and their ITAFs as a strategy to inhibit tumour growth [114]. For instance, knowing that HCV infection is a major cause of the development of hepatocellular carcinoma, strategies targeting *HCV* IRES are believed to represent a potential strategy for cancer prevention and treatment [114,133].

### 4.2. IRESs as Tools

Apart from being desirable targets for new therapeutical approaches, IRES structures can also be used as tools for the design of new therapies (Figure 3B). In 1994, Zitvogel and colleagues [134] developed a vector co-expressing the biologically active human interleukin 12 subunits and the resistance gene to neomycin. This was the first biomedical use of IRES in a bicistronic expression vector [134]. Over the past decades, there have been several studies validating this concept of using vectors to simultaneously express two genes, with therapeutic benefits in various animal models in the field of cardiovascular diseases and cancer [135,136,137], including a bicistronic IRES-based vector assessed in a clinical assay of gene therapy on patients with refractory coronary disease that co-expressed FGF2 and VEGF-A [138]. Douin and colleagues have constructed tricistronic retroviral vectors with IRESs to express different proteins from the same mRNA, specifically CD70 and CD80, two co-stimulatory molecules that can induce an antitumour response in syngeneic mice, and obtain genetically modified melanoma cells [139]. In 2012, a screening of anti-AD agents, to check the effects of curcumin and demethoxycurcumin on the IRES of APP and tau protein, originated an assay system using a bicistronic reporter construct expressing both proteins [140]. Recently, Faisal et al. tailored the size of the intercistronic spacer sequence at the 5′ end of an IRES sequence in a bicistronic vector optimised for gene therapy of familial hypercholesterolaemia [141]. IRES-based vectors have now proved their safety and therapeutic capacity, receiving the deserved validation for their use in pre-clinical and clinical studies. In addition, stress-dependent IRES activation, accounts for promising vector improvements, resulting in more efficient gene therapy [142]. Over the past few years, the potential of IRESs as biomedical tools has increased and, therefore, they have been used in combined gene therapy [142]. It is important to consider the tissue specificity when choosing an IRES, avoid systematically using a generic IRES like *EMCV* IRES, and also evaluate how the regulation and efficiency of IRES activity can be affected by the microenvironment [142]. IRES activity has been particularly documented in the field of cardiovascular diseases and cancer, showing therapeutic benefits obtained in different animal models. Some examples include the combined expression of VEGF-A and PDGFB to induce therapeutic angiogenesis in the ischemic leg and heart, and, in the case of rare diseases, the co-expression of microdystrophin and IGF1 from two adeno-associated viral (AAV) vectors to increase muscle mass and strength, decrease myofibre degeneration, and improve protection against contraction-induced injury in muscular dystrophy X-linked (MDX) mice [142]. Modified RNA has also shown therapeutic utility in IRES-associated diseases, as is the case of a synthetic modified RNA (modRNA) encoding VEGF-A in mice suffering from myocardial infarction [143]. Contrary to the control group, mice injected with *Vegfa*-modRNA into the myocardium had a reduced infarct size, improved cardiac function, and prolonged long-term survival [143]. Mice suffering from hypoxia-induced cardiomyocyte apoptosis have also been treated with modRNA technology to deliver insulin-like growth factor 1 (IGF1) into their hearts, to assess its potential cytoprotective effects [144]. Administration of *Igf1*-modRNA led to an increase in IGF1 secretion, which, in turn, improved cardiomyocyte survival under hypoxia [144,145]. Shaimardanova et al. reviewed the therapeutic use of multicistronic vectors in the expression of proteins lacking in several conditions [146]. Here, we summarise the proteins whose expression is enhanced using an IRES-based multicistronic vector and what the outcome of their use is in some of the conditions listed in Section 3 (Table 2).

#### 4.2.1. Parkinson’s Disease

Parkinson’s disease is caused by the reduction of dopamine levels as a consequence of cell death of the neurons producing this neurotransmitter [159]. Thus, because foetal dopaminergic transplants in the striatum have shown to be efficient therapies, Azzouz et al. developed a dopamine replacement gene therapy approach for PD, using a lentiviral vector system [147]. They have used a self-inactivating (SIN) tricistronic equine infectious anaemia virus (EIAV) vector genome encoding the three genes needed for the synthesis of dopamine—aromatic amino acid dopa decarboxylase (*AADC*), tyrosine hydroxylase (*TH*), and GTP cyclohydrolase 1 (*CH1*)—linked by two IRESs in a single transcription unit [147]. Injection of this vector in the rat striatum led to transduction and consequent long-term expression of the three needed enzymes in the striatum, as well as an effective production of catecholamines, and a significant reduction in motor asymmetry [147]. The resulting EIAV vector could, therefore, correct a model of PD using a dopamine replacement approach [147]. This was then taken into phase I/II clinical trials under the name ProSavin, which has been proven to be well tolerated with good signs of efficacy [148]. A continued evaluation of these patients demonstrated ProSavin as a safe and efficient treatment for PD patients for up to eight years [149]. On another note, oral administration of 3,4,-dihydroxyphenylalanine (L-DOPA) has been used as a common treatment for PD, although its effectiveness varies among patients and decreases over time [160]. A self-inactivating retrovirus (pSIR) vector was constructed to drive the expression of a bicistronic sequence containing the genes for human TH and rat GTP cyclohydrolase I (GC) separated by an IRES [150]. Marrow stromal cells (MSCs) transduced with the pSIR containing the phosphoglycerate kinase-1 (PGK) promoter were able to synthesise and secrete L-DOPA and maintain its production for three to four weeks [150]. These two examples present themselves as successful uses of IRESs as tools to resume the expression of proteins required for a healthy phenotype in PD patients. 

#### 4.2.2. Diabetes

Type I diabetes mellitus (T1D) is an autoimmune disease characterised by the destruction of β cells in the islets of Langerhans, for which the main therapeutic strategies have focused on whole pancreas transplantation or the introduction of new islets into the portal vein [161]. A caveat of such an approach is the lack of pancreas donors and the immune rejection against islets [161]. Induced pluripotent stem cells (iPSCs) can be a source of insulin-producing cells [162]. The transcription factors pancreatic and duodenal homeobox-1 (PDX-1), neurogenic differentiation 1 (NeuroD1), and V-maf musculoaponeurotic fibrosarcoma oncogene homolog A (MafA) are crucial for pancreatic β cells differentiation and function, as the three together significantly boost insulin secretion [163]. Thus, Wang et al. constructed a multicistronic adenoviral vector able to reprogramme hepatocytes into insulin-producing cells, so as to correct the diabetic state in vivo [151].

#### 4.2.3. Fabry Disease

Fabry disease results from the deficiency in the enzyme human α-galactosidase A (α-Gal A). Retroviral bicistronic vectors that allow coexpression of drug-selectable markers alongside non-selectable genes have been used as therapeutical approaches to Fabry disease [152]. Sugimoto et al. constructed a retroviral bicistronic vector containing the human multidrug-resistant gene (*MDR1*) as the first cistron and the *α-Gal A* cDNA as the IRES-translated cistron, demonstrating the efficient coexpression of the two transduced genes as gene therapy for Fabry disease [152]. Later, a more suitable Fabry murine model was generated that supports human haematopoiesis, in a study that provides crucial preclinical data for a Fabry gene therapy based on the use of IRES-containing bicistronic vectors [164].

#### 4.2.4. Mocupolysaccharidosis III A

Mucopolysaccharidosis type IIIA, a severe degenerative disease, is caused by an autosomal recessive defect of a gene encoding a lysosomal heparan-N-sulfamidase, the N-sulfoglycosamine sulfohydrolase (SGSH), whose catalytic site is activated by a sulfatase-modifying factor (SUMF1). Diagnosed children were submitted to intracerebral injections of an adeno-associated viral vector serotype, rh.10-SGSH-IRES-SUMF1 vector, in phase I/II clinical trial and it was observed an improvement in behaviour, attention, and sleep [153]. This is a well-succeeded example of a bicistronic vector used to resume the regular protein levels needed to develop a healthy phenotype. Expression of both proteins can occur simultaneously due to an IRES element upstream of the second cistron’s ORF.

#### 4.2.5. Autoimmune Diseases

A complex dysregulation of the immune system is the basis of autoimmune diseases. Interleukin (IL)-27 regulates autoimmune diseases by suppressing T helper17 (Th17) and IL-17 [165]. A study on adipose-derived mesenchymal stem cells (AD-MSCs) created genetic engineered MSCs to release IL-27, so that they could be used for reduction of inflammation and, ultimately, as therapy in autoimmune diseases. [154]. These authors transduced MSCs with a pCDH-CMV-p28-IRESEBI3-EF-copGFP-Pur lentiviral vector and evaluated IL-27 by IL-10 expression, showing that the lentiviral vector led to an increased expression of IL-27 and consequent IL-10, an anti-inflammatory cytokine, production, with no impact on MSC characteristics [154]. Since dysregulation of IL-10 is associated with an increased risk of developing many autoimmune diseases, the ability of this system to correct the functionality of IL-27 and subsequent IL-10 expression levels [166] is a promising therapeutic approach to treat several autoimmune diseases.

#### 4.2.6. Cardiovascular Diseases

The correct levels of growth and angiogenic factors expression are of the utmost importance in the maintenance of muscles, including cardiac muscles and vessels. Many cardiovascular diseases arise due to the lack of some of these factors. Several studies so far have shown the use of multicistronic IRES-based vectors in the correction of the expression levels of proteins needed to prevent such diseases. An IRES-based bicistronic vector expressing two angiogenic factors, FGF2 and cysteine-rich angiogenic inducer 61 (Cyr61) was developed and electrotransferred into a hindlimb ischemic muscle mouse model, and shown to give a more stable expression than a monocistronic plasmid [155]. Interestingly, although the bicistronic system produces 5–10 times less of each angiogenic molecule than the monocistronic ones, it promotes more abundant and functional revascularization than the latter [155]. These results validate the use of IRES-based bicistronic vectors for the coexpression of monitored low doses of therapeutic molecules, as they show active cooperation of FGF2 and Cyr61 in therapeutic angiogenesis of hindlimb ischemia, providing a safe gene therapy [155]. The therapeutic potential of adeno-associated virus (AAV)-mediated expression of VEGF and bone morphogenetic protein (BMP) has also been investigated as a new therapeutic technique for the treatment of avascular necrosis of the femoral head [156]. In the rabbit ischemic hind limb model infected with the lentiviral construct rAAV-hVEGF165-IRES-hBMP-7 (AAV-VEGF/BMP), the levels of VEGF165 and BMP-7 increased over time and resulted in a stronger osteogenic ability than the control counterpart. The result was an orthotopic ossification, capillary growth, and calcium deposit formation [156]. A similar approach using an AAV multicistronic vector containing two angiogenic genes (AAV-FGF4-IRES-VEGF-A) has been used to improve recovery from acute limb ischemia [157]. Simultaneous expression of VEGF-A and FGF4 stimulated the remodelling of the capillary wall in the non-ischemic model and increased the number of capillaries in normo-perfused hindlimbs [157]. Furthermore, concurrent expression of both factors restored the post-ischemic foot blood flow faster than the control and decreased toe necrosis [157]. In another example, to assess the effect of simultaneous expression of two proteins that synergistically promote angiogenesis, stromal cell-derived factor 1 (SDF-1) and VEGF proteins, an AAV expressing *VEGF165* and *SDF-1* connected via an IRES was used in a rat model of cerebral infarction [158]. The vector allowed coexpression of both proteins in multiple locations around the ischemic core and contributed to several aspects, such as neural function, cerebral infarction volume reduction, microvascular density increase, angiogenesis stimulation in the ischemic penumbra and cerebral blood flow and perfusion, which may be a useful approach for improving vascular reshaping and regaining of neural function after cerebral infarction [158].

## 5. Conclusions

Precision medicine is evolving towards the use of specific treatments for a given genetic condition. The use of RNA-based therapies is a growing field of evidence and practical application in precision medicine. Understanding the mechanisms associated with conditions generated by dysregulation of IRES-mediated translation, and/or their controlling ITAFs is of great importance to developing new medicines and contributing to the progress of precision medicine. The use of ASOs is becoming more ordinary and effective, as mainly they are becoming harmless and free of side effects. Also, the fact that several transcripts are polycistronic opened the way to manufacturing polycistronic vectors to account for the co-expression of different proteins according to the needs imposed by a given condition. These are two approaches that converge on the idea of developing precise medicines, using IRESs either as targets or tools (Figure 3). Although there is still a long way to go in the era of personalised medicine by using IRES either as targets or tools, the already available, and the putative new therapeutic approaches that are emerging are a significant promise to treat and minimise the suffering of many patients.

## Figures and Tables

**Table 1:** Summary of different groups of diseases, other than cancer, caused by IRES-mediated translation initiation misregulation. Here are listed the internal ribosome entry sites (IRESs) and IRES *trans*-acting factors (ITAFs) correlated to each pathology. The existing IRES-related RNA-based therapies for each pathology are also included.

	Pathologies	IRES-Containing Transcripts	Related ITAFs	Tested RNA-Based Therapies	References
Neurodegenerative diseases	Spinocerebellar ataxia type 6	*CACNA1A*	n.i. *	miRNA-based therapy	[65,66]
	Fragile X syndrome	*fmr1*	hnRNPQ	n.i. *	[70]
	Alzheimer’s disease	*p53* (*p44 isoform*)	APP (AICD), nucleolin	n.i. *	[74]
	Parkinson’s disease	*HIF-1α*	PINK1	Antisense oligo nucleotide reducing the expression of α-synuclein pathogenic protein	[82,83]
	Amyotrophic lateral sclerosis		Related RBPs: hnRNPA2/B1, hnRNPA1, FUS	n.i. *	[84]
Muscular atrophies	Ischemic cardiomyopathy	*VEGFA*, *VEGFC*, *FGF1*	hnRNPL, VASH1	n.i. *	[90,91]
	Myogenesis regulation	*FGF1*/*FGF2*	hnRNPM, p54^nrb^	n.i. *	[95,110]
	Duchenne muscular dystrophy	*utrophin A*	eEF1A2	IRES over-expression by small molecules	[102,111]
Other diseases	Diamond-Blackfan anaemia	*Bag1*/*Csde1*, *p53*	Rps19, Rpl11	n.i. *	[107]
	Diabetes	*INR/IGF-1R*	PTBP1, HuR, hnRNPC	miRNA-based therapy	[108,109]

* n.i.: no information available.

**Table 2:** Summary of diseases/conditions that benefit from the use of IRES-based multicistronic vectors co-expressing different proteins to restore the expression levels of proteins required to resume the wild-type phenotype.

Disease/Condition	IRES Gene Therapy	Expressed Proteins	Purpose	References
Parkinson’s disease	Multicistronic lentiviral construct Bicistronic retroviral construct	Tyrosine hydroxylase (TH) Aromatic amino acid dopa decarboxylase (AADC) GTP cyclohydrolase 1 (CH1) human TH, rat GC	Dopamine synthesis Synthesis of L-DOPA	[147,148,149,150]
Diabetes	Multicistronic adenoviral construct	Pancreatic and duodenal homeobox-1 (Pdx1), Neurogenin 3 (Ngn3) V-musculoaponeurotic fibrosarcoma oncogene homolog A (MafA)	Reprogramming of hepatocytes into insulin-producing cells in vitro and correcting the diabetic state in vivo	[151]
Fabry disease	Bicistronic retroviral vectors	a-Gal A gene drug-selectable multidrug resistance gene 1 (MDR1)	Restore the deficiency of the α-galactosidase A (a-Gal A) enzyme	[152]
Mucopoly- saccharidosis IIIA	Adeno-associated virus (AAV)-based bicistronic vector	Heparan-N-sulfamidase and N-sulfoglycosamine sulfohydrolase (SGSH) Sulfatase-modifying factor (SUMF1)	Improve heparan sulfate catabolism and decrease microglial activation	[153]
Autoimmune diseases	Bicistronic lentiviral vector	Two IL-27 subunits (p28 and EBI3)	Promote the differentiation of T-cells that secrete IL-10	[154]
Cardiovascular diseases	Multicistronic vectors Bicistronic IRES-based AAV vector Other IRES-based multicistronic vectors	FGF2 Cysteine-rich angiogenic inducer 61 (Cyr61) VEGF Bone morphogenetic protein (BMP) VEGF165/stromal cell-derived factor-1 (SDF-1)	Formation of a new vascular network in the hindlimb ischemia mouse model Genetic modification of rabbit bone marrow-derived mesenchymal stem cells Effective in therapy for ischemia animal models in vivo	[155,156,157,158]
